# A Dual-Modal Robot Welding Trajectory Generation Scheme for Motion Based on Stereo Vision and Deep Learning

**DOI:** 10.3390/ma18112593

**Published:** 2025-06-01

**Authors:** Xinlei Li, Jiawei Ma, Shida Yao, Guanxin Chi, Guangjun Zhang

**Affiliations:** 1 State Key Laboratory of Precision Welding & Joining of Materials and Structures, Harbin Institute of Technology, Harbin 150001, China; lixinlei@hit.edu.cn (X.L.); mjw_1103@163.com (J.M.); 24s009069@stu.hit.edu.cn (S.Y.); 2 School of Mechanical and Electrical Engineering, Harbin Institute of Technology, Harbin 150001, China; chigx@hit.edu.cn

**Keywords:** dual-modal perception, intelligent weld recognition, multi-layer, multi-bead path planning, U-Net

## Abstract

To address the challenges of redundant point cloud processing and insufficient robustness under complex working conditions in existing teaching-free methods, this study proposes a dual-modal perception framework termed “2D image autonomous recognition and 3D point cloud precise planning”, which integrates stereo vision and deep learning. First, an improved U-Net deep learning model is developed, where VGG16 serves as the backbone network and a dual-channel attention module (DAM) is incorporated, achieving robust weld segmentation with a mean intersection over union (mIoU) of 0.887 and an F1-Score of 0.940. Next, the weld centerline is extracted using the Zhang–Suen skeleton refinement algorithm, and weld feature points are obtained through polynomial fitting optimization to establish cross-modal mapping between 2D pixels and 3D point clouds. Finally, a groove feature point extraction algorithm based on improved RANSAC combined with an equal-area weld bead filling strategy is designed to enable multi-layer and multi-bead robot trajectory planning, achieving a mean absolute error (MAE) of 0.238 mm in feature point positioning. Experimental results demonstrate that the method maintains high accuracy under complex working conditions such as noise interference and groove deformation, achieving a system accuracy of 0.208 mm and weld width fluctuation within ±0.15 mm, thereby significantly improving the autonomy and robustness of robot trajectory planning.

## 1. Introduction

With the rapid development of intelligent manufacturing, traditional welding robot methods such as teaching playback and offline programming no longer meet high-precision requirements under dynamic conditions. These methods are plagued by issues such as weak environmental perception and poor real-time adjustment [1,2,3], as they rely on manually preset paths. For instance, in curved or multi-layer welding, workpiece deformation and heat input often cause trajectory errors, which necessitate manual corrections and negatively impact efficiency and quality [4]. Consequently, visual autonomous programming methods integrating multimodal perception and high-precision positioning have emerged as a focal point. These methods can provide real-time weld morphology and spatial posture data for dynamic robot path planning, thereby facilitating the advancement of intelligent welding.

Current solutions can be categorized into two primary types: local detection employing line-structured light and global reconstruction utilizing stereo vision [5,6]. The former exhibits high accuracy (±0.1 mm) but demonstrates low scanning efficiency and limited adaptability to complex paths [7]. By contrast, the latter employs multi-view point cloud reconstruction to efficiently obtain full 3D weld information, thereby enabling automatic weld recognition and autonomous path planning. Consequently, research has centered on point cloud processing and deep learning.

Many scholars have explored weld seam recognition through point cloud processing. Zhou et al. [8] introduced a robotic arc welding path planning system integrated with an RGB-D camera controlled via gesture-based interaction. Their approach employed iterative closest point (ICP) for 3D model reconstruction and an extended bilateral filter for noise reduction. Using edge strength-based methods, they extracted weld features and generated a smooth welding path with posture optimization. Experimental results demonstrated robust performance across diverse workpieces, achieving a matching accuracy exceeding 94%. Zhang et al. [9] proposed a weld tracking method utilizing laser triangulation and a systematic four-step process. Following filtering and noise reduction, weld corner points were identified via a second-order derivative algorithm, and positional deviations were addressed through linear fitting. This methodology achieved a 38–42% reduction in positioning errors, satisfying the accuracy requirements for industrial welding applications. Automated defect recognition methods, such as the X-ray image processing technique developed by Chi et al. [10], provide a complementary approach to validate the robustness of weld morphology extraction in complex joint configurations.

In recent years, deep learning-based weld recognition methodologies have garnered significant attention owing to their high precision and robust performance. Convolutional neural networks (CNNs) have notably advanced weld detection by automating the extraction of multi-scale features, driving methodological innovation in the field. Lei et al. [11] proposed a novel method for multi-layer, multi-pass weld bead cross-section morphology extraction, leveraging row–column grayscale segmentation to enhance the precision of weld geometry characterization. This approach aligns with the requirements of multi-bead trajectory planning by enabling accurate cross-sectional area adaptation, which is critical for maintaining consistent weld formation in layered welding scenarios. Zhang et al. [12] demonstrated the effectiveness of deep learning in detecting defects during keyhole TIG welding, utilizing enhanced vision systems to improve detection accuracy under high-temperature and high-noise conditions. Their work underscores the importance of integrating advanced vision technologies for real-time quality monitoring, which complements the robustness requirements of the proposed dual-modal framework.

Current investigations into robot welding trajectory planning indicate that point cloud processing-based approaches are capable of extracting weld features in the 3D space; however, they are frequently constrained by the sensor’s broad acquisition scope and inherent object complexity. The resultant point cloud often contains substantial redundant and irrelevant information, thereby reducing processing efficiency and leading to unstable recognition performance due to interference points. Conversely, while deep learning-based methods exhibit robustness and accuracy in 2D image recognition tasks, they fall short in providing precise 3D spatial positioning, thereby limiting their ability to directly facilitate accurate welding path planning. Recent advancements in penetration state recognition, such as the two-stage temporal convolutional network proposed by Liu et al. [13], have demonstrated significant potential for real-time monitoring of welding quality, particularly in high-precision applications like laser welding.

**Figure 1:**
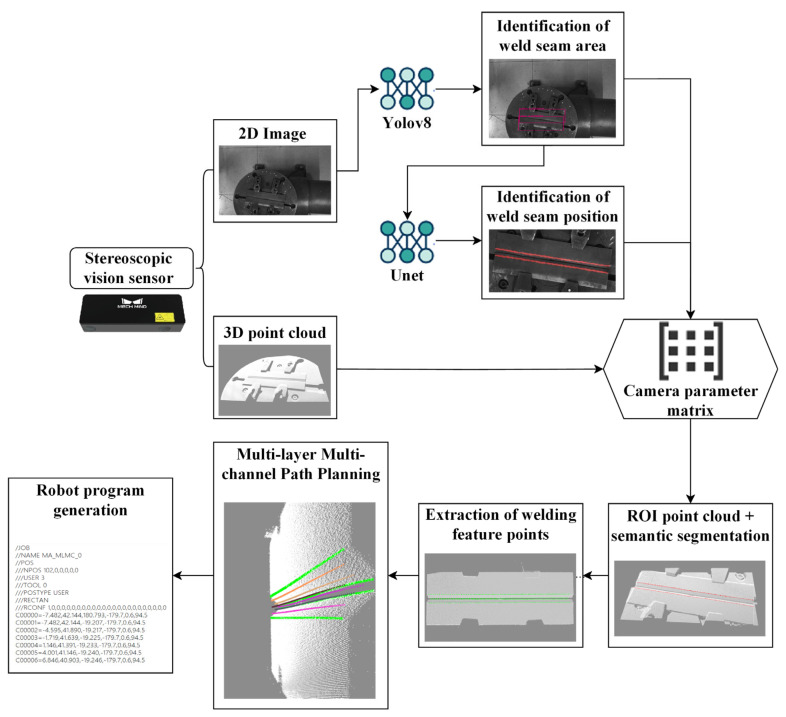
Overall process.

To address these challenges, this paper focuses on robot welding trajectory generation, investigating welding robots equipped with stereo vision systems, and presents a dual-modal collaborative framework termed “2D image autonomous recognition + 3D point cloud precise planning”, the overall process of the algorithm is shown in Figure 1. For 2D image recognition, the YOLOv8s model is employed to enable rapid localization of the welding plate’s region of interest (ROI). Pixel-level weld segmentation is achieved through the enhancement of a U-Net deep learning model, after which 2D semantic information is mapped to 3D point cloud space thereby achieving point cloud segmentation. Conversely, while deep learning-based methods exhibit robustness and accuracy in 2D image recognition tasks, for 3D point cloud planning, a multi-layer, multi-bead path planning algorithm based on hierarchical slicing and feature mapping is developed to enable precise robot trajectory planning within 3D point cloud environments. This approach effectively overcomes the technical bottlenecks of high computational complexity and limited environmental adaptability inherent in traditional point cloud processing methods, thereby improving the autonomous operational capability of robots in welding scenarios. The proposed framework contributes to enhancing the intelligence of welding robots and promoting the intelligent evolution of industrial welding processes.

## 2. Key Technologies

### 2.1. Research on Intelligent Weld Recognition

In this study, we present a weld recognition framework that integrates the YOLO algorithm and an enhanced U-Net deep learning model. The methodology comprises three critical stages: First, upon acquiring weld images with complex backgrounds, YOLO is employed to accurately localize the weld plate region of interest (ROI), thereby mitigating computational redundancy and misidentification risks arising from whole-image feature extraction. Second, to address surface interferences such as occlusion and oxidation on the parent material, an attention mechanism is integrated into the U-Net architecture, enabling pixel-level precise segmentation of weld regions. Finally, a rigid calibration-based cross-modal information association method is designed, which performs dynamic equidistant sampling on segmentation results, establishes bidirectional mappings between 2D pixel coordinates and 3D point cloud coordinates, enhances path smoothness and spatial alignment accuracy, and provides foundational support for multi-layer, multi-bead welding path planning.

#### 2.1.1. YOLOv8 Weld Area Recognition Preprocessing

When the system captures a weld image with a complex background, the initial stage in generating the robot welding trajectory entails autonomous recognition and localization of the weld region. This study employs a YOLO-based target detection algorithm to accurately locate the weld plate area, ensuring efficient spatial confinement for subsequent processing.

YOLOv8, a state-of-the-art target detection framework grounded in convolutional neural networks [14,15,16,17], involves several key stages during training: dataset compilation, image annotation, dataset partitioning, hyperparameter configuration, and iterative validation of model performance. The compilation and annotation of datasets serve as foundational pillars for training the YOLOv8 weld plate recognition model, as the quantity and quality of these datasets directly influence the precision and reliability of downstream models.

In this research, 1060 images of weld plates with varying dimensions, acquired under diverse lighting conditions via a 3D visual sensor, were systematically collected. Through the application of geometric transformations (rotation and mirroring), noise perturbations (Gaussian/salt-and-pepper noise and brightness adjustments), and blur processing (motion blur and out-of-focus blur), alongside additional augmentation strategies, the dataset was expanded to 10,600 images. The dataset was partitioned into training, validation, and test subsets at a ratio of 70%, 15%, and 15%, respectively, to ensure robust model generalization.

**Figure 2:**
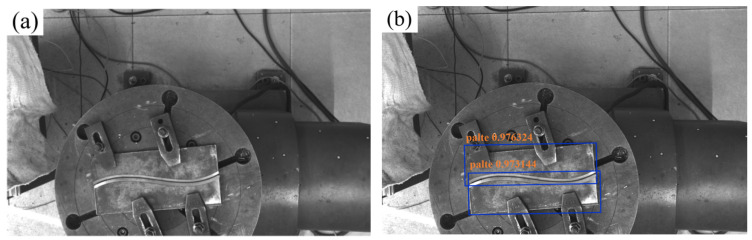
YOLOv8 model prediction results. (**a**) Original image; (**b**) predicted image.

The YOLOv8 model was trained utilizing the PyTorch 1.10.1 deep learning framework. The host system, equipped with an NVIDIA RTX 3070 GPU, leveraged the NVIDIA CUDA toolkit to accelerate both training and inference processes, optimizing computational efficiency. The trained model is capable of automatically localizing the weld plate within an input image, as illustrated in Figure 2. Phase contrast imaging techniques, as explored by Yang et al. [18], offer enhanced resolution for micro-gap weld seam detection, which could further refine the precision of the proposed stereo vision system.

**Figure 3:**
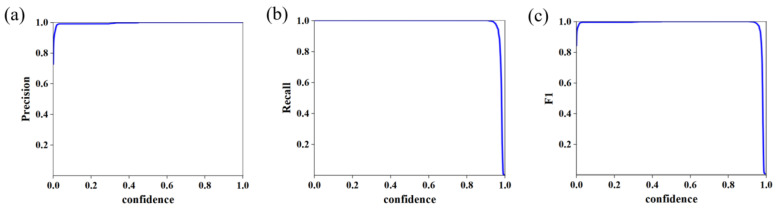
YOLOv8 model training results. (**a**) P_curve; (**b**) R_curve; (**c**) F1_curve.

**Figure 4:**
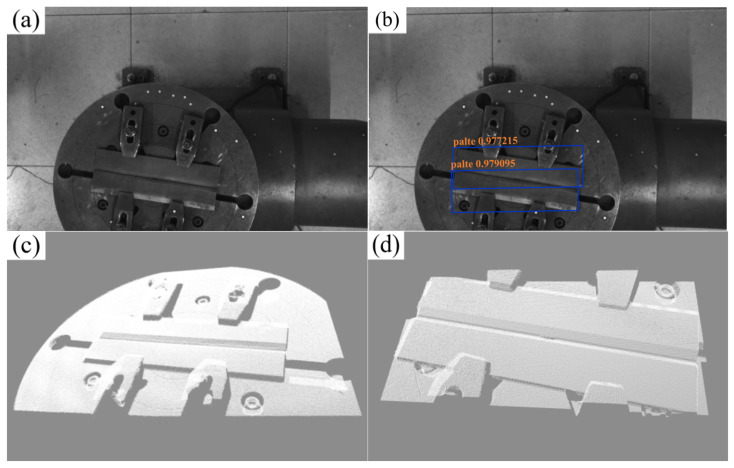
YOLOv8 ROI welding plate point cloud recognition process. (**a**) Weld image; (**b**) YOLO recognition result; (**c**) overall point cloud; (**d**) weld area point cloud.

As depicted in Figure 3, the model training curve illustrates the convergence of key performance metrics. The trained model achieved 100% accuracy and recall on the test set, alongside a bounding box loss (box_loss) of 0.26%, demonstrating exceptional detection precision. These results indicate the model’s capability to accurately localize and determine the 2D coordinates of weld plates within complex background images. By integrating camera calibration parameters, the corresponding 3D coordinates of 2D pixel points are systematically calculated, enabling precise extraction of the 3D point cloud region of interest (ROI) for weldments. As demonstrated in Figure 4, the outcomes of weldment recognition and point cloud extraction validate the framework’s effectiveness in bridging 2D image semantics and 3D spatial information.

#### 2.1.2. Improvement and Training of U-Net Model for Weld Position Recognition

Following the extraction of the weld plate region of interest (ROI) via the YOLO algorithm, this paper presents an enhanced U-Net-based weld recognition approach to address the challenge of diminished image recognition accuracy caused by surface interferences—such as occlusion and oxidation—on the base material.

U-Net is a well-established semantic segmentation network [19], and its core architecture is an encoder–decoder structure that balances both global semantics and local details. Although U-Net performs well in common applications such as medical image segmentation, it exhibits the following limitations in industrial scene image segmentation:

First, the original U-Net employs a shallow encoder without a robust backbone network, resulting in inadequate feature representation for high-resolution images with complex weld textures. This limitation hinders the accurate identification of fuzzy boundaries and high-frequency details critical for precise segmentation. Backbone networks such as VGG16, which demonstrate proficiency in semantic extraction and multi-layer feature expression, address this gap by enhancing hierarchical feature learning and recognition accuracy. Second, the absence of an attention mechanism in the standard U-Net architecture may lead to blurred object boundaries and target misclassification, particularly in noisy industrial environments. By integrating the DAM module [20], which incorporates channel and spatial attention mechanisms, the model’s capability to prioritize discriminative local features is strengthened, enabling more accurate delineation of weld regions. Additionally, reliance on a single loss function in conventional U-Net implementations renders it ineffective in addressing challenges such as category imbalance, edge localization errors, and inconsistent regional segmentation. To mitigate these issues, this study combines Dice Loss [21], Focal Loss [22], and boundary refinement. These modifications enhance both segmentation accuracy and the model’s generalization capacity across diverse industrial welding scenarios.

**Figure 5:**
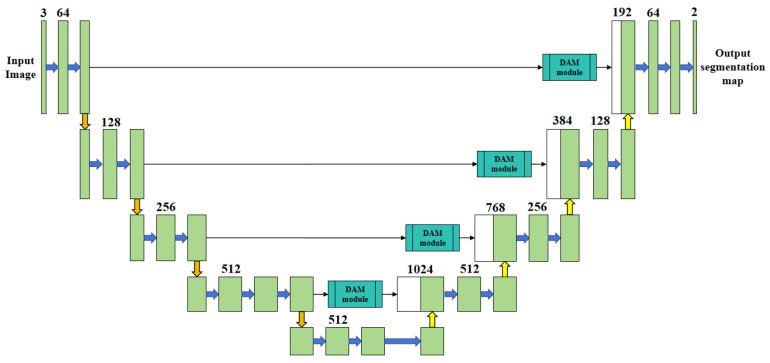
The network structure of improved U-Net.

To address these issues, based on the U-Net framework, this paper proposes an improved model. This model employs VGG16 as the encoder’s backbone network, incorporates the DAM attention module, and combines Dice Loss, Focal Loss, and Weighted IoU Loss [23] for joint training (as shown in Figure 5). The model is developed with the aim of enhancing the accuracy and robustness of weld segmentation.

In the backbone network, VGG16 substitutes the original encoder module to enhance the feature extraction capabilities and model generalization performance. The structure is composed of five convolution blocks that form a multi-scale feature pyramid, with the number of channels increasing from 64 to 512. The input image is downsampled from 512 × 512 × 3 to 32 × 32 × 512, enabling the extraction of features ranging from shallow-level texture details to deep-level structural information. The shallow layers are dedicated to capturing texture features, the middle layers concentrate on detecting directional cues, and the deep layers emphasize the extraction of edge and topological features, which enhances the recognition of complex welds. The multi-layer fusion refines edge and spatial details, thereby boosting the accuracy of weld stripe recognition.

During the model enhancement phase, this study introduces a dual-channel attention module (DAM) into the enhanced U-Net architecture to improve the robustness of weld recognition under complex backgrounds. The DAM module incorporates both channel and spatial attention mechanisms to strengthen feature representation capabilities, enabling the network to better handle hierarchical visual information. Specifically, the module is embedded within cross-connection layers to dynamically enhance features during high-level semantic and low-level detail feature extraction stages. Channel attention enhances the activation of semantically relevant channel features, thereby boosting discriminative power for distinguishing between different weld types. Spatial attention, conversely, enhances the spatial consistency of target regions and mitigates background interference, improving the localization of weld boundaries. Through weighted fusion of these two attention mechanisms, the proposed approach effectively enhances both weld segmentation accuracy and boundary positioning precision without imposing substantial increases in model computational overhead. Experimental results indicate that this method exhibits superior robustness, particularly in challenging conditions involving occlusion, scale variations, and noise interference.

In the loss function design stage, based on the task characteristics of weld identification and positioning, a joint loss strategy (Dice–Focal–Weighted IoU Loss) is proposed, integrating Dice Loss, Focal Loss, and Weighted IoU Loss. This strategy aims to enhance model performance in three aspects: regional consistency, category balance, and edge accuracy.

(1) Dice Loss to improve the overall consistency of the weld area

Dice Loss effectively alleviates the problem of imbalance between the weld area and the background and improves the segmentation accuracy by maximizing the overlap between the prediction and the true label. It is defined as follows:

(1) $$L_{Dice}=1-\frac{2\sum_{i}p_{i}g_{i}+\in }{\sum_{i}p_{i}^{2}+\sum_{i}g_{i}^{2}+\in }$$

where $p_{i}$ and $g_{i}$ are the predicted probability and true label of the *i*-th pixel, respectively, and ∈ is a smoothing factor (usually 10^−6^ to prevent the denominator from being zero). By maximizing the Dice coefficient, the model can enhance the overall modeling ability of the weld contour while maintaining the integrity of the region.

(2) Focal Loss to solve the problem of imbalance between weld and background categories

Since the weld area accounts for a small proportion of the image, it is easily affected by category imbalance during training, resulting in incomplete recognition. Focal Loss can improve the model’s ability to learn weld edges and complex shapes by increasing the loss weight of difficult-to-classify samples, thereby improving the model’s ability to extract weld areas under complex backgrounds. Its definition is as follows:

(2) $$L_{Focal}=-\alpha {\left(1-P_{t}\right)}^{\gamma }\log\left(P_{t}\right)$$

where $P_{t}$ represents the predicted probability of the positive sample, α is the balance factor (usually 0.25), and γ is the adjustment factor (usually 2).

(3) Weighted IoU Loss to enhance the positioning accuracy of weld edges

In weld recognition, edge positioning accuracy directly affects the path planning effect. Traditional IoU Loss has shortcomings in edge modeling. This paper introduces Weighted IoU Loss. By giving higher weights to edge pixels and enhancing edge feature learning, the model can more accurately identify and locate weld boundaries and improve segmentation accuracy under complex weld morphology. Its definition is as follows:

(3) $$L_{IoU}=1-\frac{\sum_{i}{w_{i}p}_{i}g_{i}\;}{\sum_{i}w_{i}(p_{i}+g_{i}-p_{i}g_{i})\;}$$

where $w_{i}$ is the dynamic weight for each pixel, which is adaptively adjusted during training based on the importance of the weld edge.

This paper adopts a weighted fusion strategy to combine the three loss functions into the final loss function:

(4) $$L_{total}=\lambda_{1}L_{Dice}+\lambda_{2}L_{Focal}+\lambda_{3}L_{IoU}$$

where $\lambda_{1}$, $\lambda_{2},$ and $\lambda_{3}$ are weight coefficients, which control the contribution of different loss terms, respectively. Through experimental adjustment, this paper sets the weight coefficients to $\lambda_{1}$ = 0.5, $\lambda_{2}$ = 0.25, and $\lambda_{3}$ = 0.25, ensuring that the detailed modeling ability of the weld boundary is enhanced while maintaining the global feature expression.

**Figure 6:**
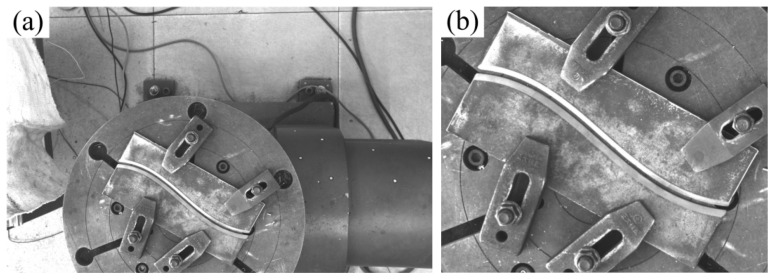
Image preprocessing. (**a**) Straight weld; (**b**) curved weld.

In dataset construction, to enhance the segmentation accuracy of the weld area for U-Net, this study introduces a model-driven preprocessing approach: the weld plate region is extracted via the trained YOLOv8s model (mAP@0.5 = 99.5%), detection boxes with confidence scores exceeding 0.85 are identified, and a 5% boundary expansion is applied to the bounding boxes before cropping. This process retains spatial contextual details (approximately 3–4 mm) around the weld, generating high-purity input data for subsequent segmentation (as shown in Figure 6). By leveraging cascaded model processing, this method effectively mitigates background interference, increases the proportion of effective pixels in the weld region, and significantly enhances the model’s capability to recognize weld features under complex background conditions.

**Figure 7:**
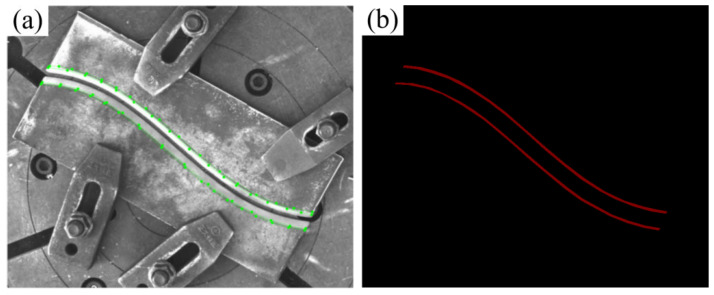
Dataset annotation. (**a**) Labeling process; (**b**) labeling results.

For the labeling process, the Labelme tool is employed to conduct precise pixel-level annotation of weld groove feature lines, with annotation errors maintained within ±2 pixels. The labeled data are then converted into VOC format mask images, which serve as training inputs for the semantic segmentation model (as illustrated in Figure 7).

**Figure 8:**
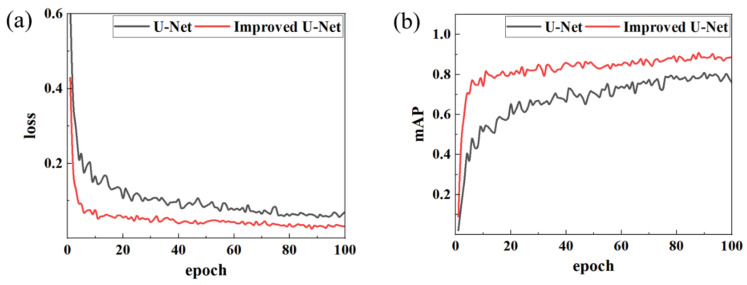
Comparison of model training results. (**a**) Loss; (**b**) mAP.

The improved U-Net model was contrasted against the original model. The Adam optimizer was employed in the training process to enhance the convergence rate. The training results are presented in Figure 8. The loss and accuracy curves of the two models exhibited favorable convergence behavior, without discernible signs of overfitting or underfitting. Moreover, the training process was stable and reliable.

Comparative analysis demonstrates that the enhanced U-Net outperforms the original model in both loss function performance and mAP metrics. The improved model’s loss value decreased to 0.09 by the fifth epoch and eventually stabilized at 0.032, a 46.7% reduction compared to the original model’s stable loss of approximately 0.06. In terms of the mAP, the enhanced model stabilized at 0.88 with a peak of 0.908, significantly surpassing the original model’s stable mAP of 0.78 (peak: 0.803). Additionally, the training curve of the improved model exhibits smoother trends with less pronounced oscillations, indicating stronger feature extraction capabilities and training stability. These results highlight that the U-Net following architectural optimizations is better adapted to local feature segmentation in weld images, achieving faster convergence and higher recognition accuracy compared to its baseline counterpart.

To comprehensively assess the improved model’s performance in weld feature recognition, this study conducts a comparative analysis between the original U-Net and the optimized model using two key metrics: mean intersection over union (mIoU) and F1-Score.

**Figure 9:**
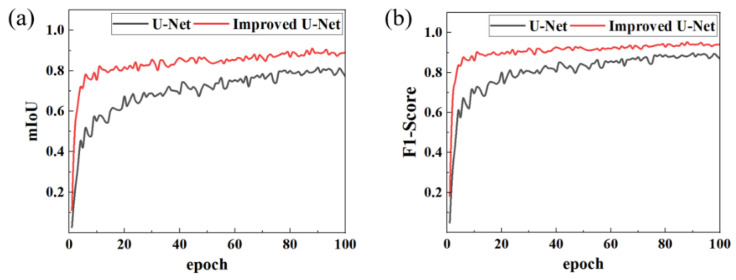
Comparison of model performance indicators. (**a**) mIoU; (**b**) F1-Score.

Figure 9 illustrates the evolution curves of key performance indicators. The results demonstrate that the optimized model exhibits substantial improvements across all metrics:

Segmentation accuracy: The mean intersection over union (mIoU) increased from 0.796 to 0.887, indicating that segmented regions align more closely with ground-truth annotations and effectively capture fine-grained weld features.

Recognition performance: Precision and recall rose to 0.937 and 0.944, respectively, significantly reducing instances of false positives (incorrect detections) and false negatives (missed detections), which highlights the model’s enhanced ability to distinguish weld regions from complex backgrounds.

Comprehensive performance: The F1-Score, a balanced measure of accuracy and robustness, improved to 0.940, reflecting the model’s superior capability to handle challenges such as occlusion and texture variation in industrial welding scenarios.

**Figure 10:**
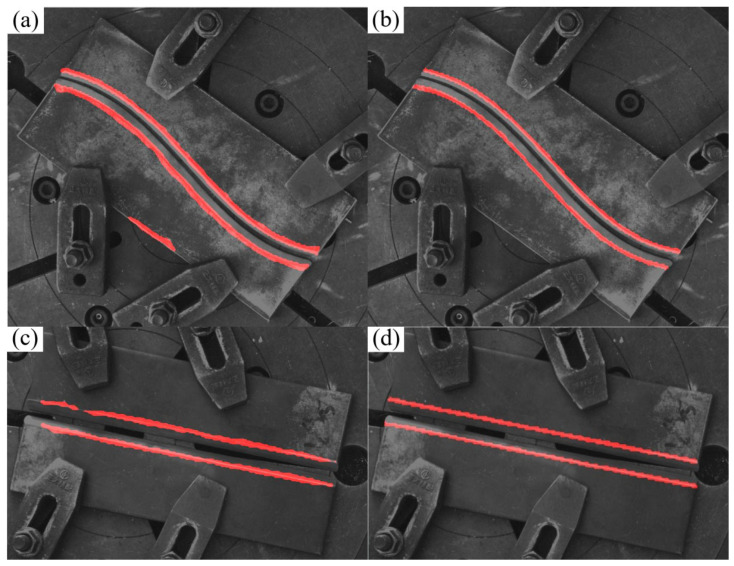
Comparison of model prediction results. (**a**) Original model prediction results (misidentification); (**b**) improved model prediction results; (**c**) original model prediction results (missed identification); (**d**) improved model prediction results.

In conclusion, the optimized U-Net model outperforms the baseline counterpart in segmentation boundary definition, detail reconstruction capability, and overall recognition accuracy, thereby validating the efficacy of incorporating the attention mechanism and implementing architectural optimizations. Figure 10 presents the model’s recognition performance in real-world industrial welding scenarios, demonstrating its practical applicability and robustness.

In addition, we also compared our model with pspNet and DeepLab V3+, two mainstream semantic segmentation task frameworks. The comparison results of the mIoU, MPA, and inference time are shown in Table 1. There are 1245 training images after data enhancement, and the training dataset and validation dataset are divided into the training dataset and validation dataset according to the ratio of 9:1. It is worth noting that since our strategy is to first use the YOLOv8 model to identify the image ROI area and then apply the improved U-Net model on the ROI area to identify the weld beam, in the training of the improved U-Net part of the model proposed in this paper, the training set first uses the Yolov8 model for target recognition and cropping, while the pspNet and DeepLab V3+ models are directly trained. In addition, the backbone feature extraction networks of U-Net, pspNet, and DeepLab V3+ are selected as VGG16, Resnet50, and Xception, respectively.

**Figure 11:**
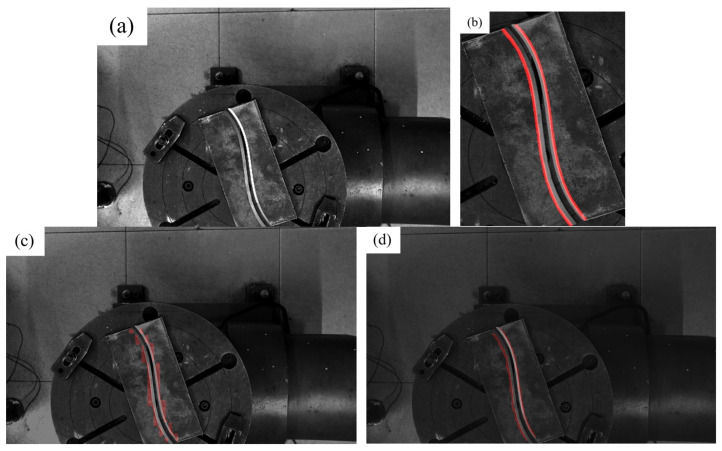
Simple scene model recognition effect. (**a**) Original image; (**b**) improved U-Net on the ROI; (**c**) pspNet (**d**) DeepLab V3+.

**Figure 12:**
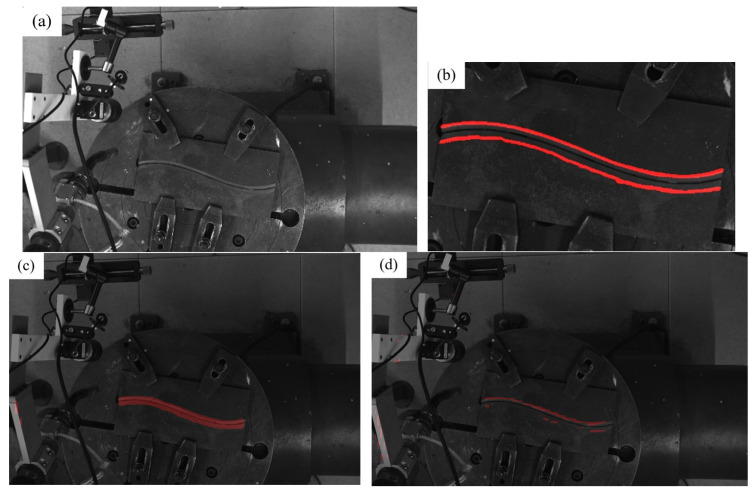
Typical complex scene model recognition effect. (**a**) Original image; (**b**) improved U-Net on the ROI; (**c**) pspNet; (**d**) DeepLab V3+.

From Table 1, we can see that the mIoU and MPA of the improved U-Net of the three models show that the improved U-Net proposed by us has a significantly better effect. However, it is worth noting that the training effect of the model is affected by many factors such as the dataset, the choice of the backbone network and loss function, the number of training rounds, and the learning rate. In addition, our improved U-Net is trained on the cropped image, so it is difficult to conclude that our improved U-Net has better performance. However, for the case where the foreground pixel ratio is small and the number of training images is limited, our strategy of combining Yolov8 and U-Net models is better in terms of algorithm stability and detection effect. As shown in Figure 11 and Figure 12, which are detection comparisons of typical simple scenes and complex scenes, respectively, it can be seen from the observation that for simple scenes, all three models can find the edge position of the weld edge, but for some objects or scenes that have not appeared in the training data images, the method without the YOLO pre-selection box is prone to misidentification.

#### 2.1.3. Extraction of Weld Skeleton Feature Points Based on Depth Image

Building on the enhanced U-Net weld segmentation results, this study presents a cross-modal weld skeleton feature extraction methodology. First, the Zhang–Suen thinning algorithm processes the segmented weld region to generate a single-pixel-width central skeleton, preserving geometric topology while reducing dimensional complexity. Next, path-length-based, equidistant, uniform interpolation samples feature points along the skeleton, ensuring consistent spatial discretization for subsequent trajectory planning. Finally, bidirectional mapping between 2D pixel coordinates (u and v) and 3D spatial coordinates (X, Y, and Z) is established to align semantic segmentation results with 3D geometric information, enabling precise weld trajectory planning by integrating visual semantics and spatial geometry.

**Figure 13:**
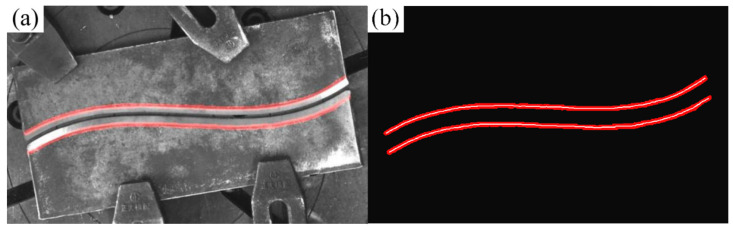
Zhang–Suen skeleton refinement effect. (**a**) Model prediction results; (**b**) skeleton refinement results.

To derive a precise weld centerline from the U-Net segmentation mask, the classical Zhang–Suen skeleton thinning algorithm is employed. This iterative technique systematically removes non-skeleton edge pixels while maintaining the image’s topological structure and connectivity, ensuring the preservation of critical geometric features. The result is a topologically simplified, single-pixel-width central skeleton that retains the complete structural integrity of the weld region. This representation is well suited for feature extraction in slender structures such as weld seams, providing an ideal foundation for subsequent geometric analysis, as demonstrated in Figure 13

**Figure 14:**
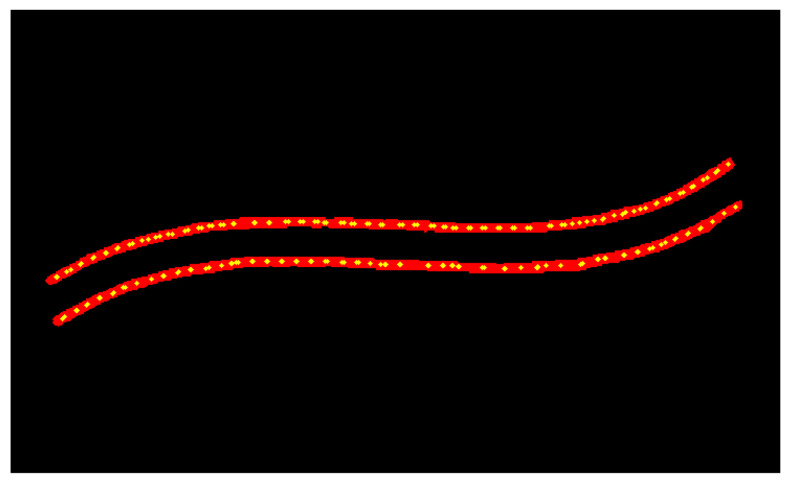
Uniform interpolation algorithm based on path length.

**Figure 15:**
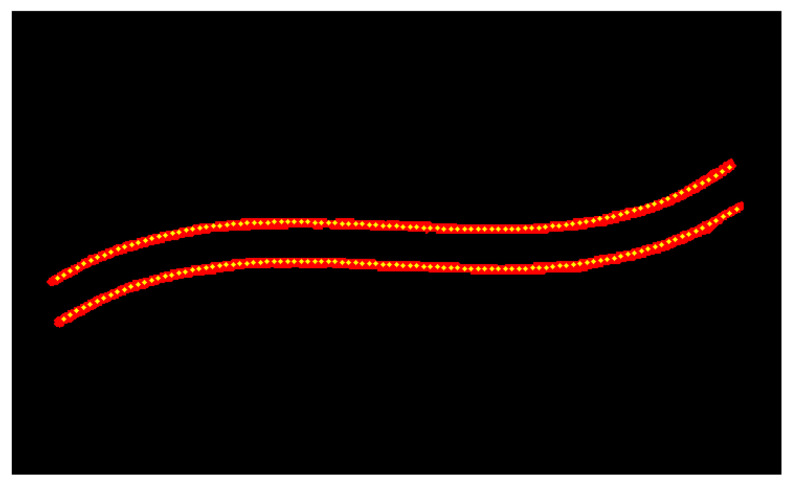
Feature point extraction algorithm based on polynomial fitting.

To extract uniformly distributed feature points along the weld skeleton, this study employs a path-length-based uniform interpolation algorithm. The algorithm first computes the total traversal length of the skeleton, then identifies sampling positions at equal intervals along this path, and finally generates feature points via linear interpolation, thereby ensuring consistent spatial distribution. To further enhance the precision and smoothness of feature point placement, a polynomial fitting approach is incorporated. Specifically, the skeleton contour is approximated using a third-order polynomial, and the fitting curve is refined via the least squares method to minimize approximation errors, yielding a smoother and more continuous sequence of feature points. As illustrated in Figure 14 and Figure 15, feature points sampled from the complexly shaped weld skeleton (depicted as green dots) closely match the original structural geometry while maintaining nearly uniform spacing. This dual-step approach—combining path-length interpolation with polynomial optimization—ensures reliable extraction of evenly spaced, geometrically consistent feature points, which are critical for subsequent trajectory planning and robotic guidance in welding applications.

Following the extraction of 2D weld skeleton feature points and the refinement of polynomial fitting, the precise mapping of 2D image semantic information to a 3D point cloud space is critical. To address this, this study employs a depth-aware mapping framework grounded in the Pinhole Camera Model, integrating camera calibration parameters to achieve coordinate conversion with sub-millimeter accuracy.

**Figure 16:**
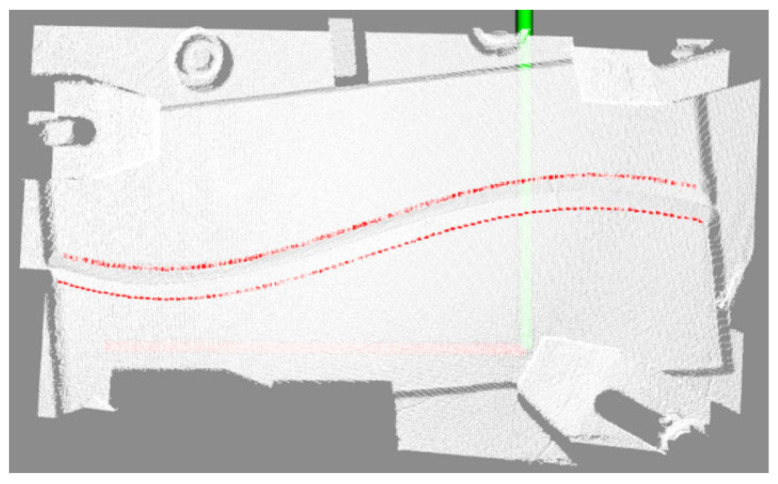
Coordinate semantic mapping results.

For the region of the depth map containing weld skeleton feature points, a 3 × 3-pixel neighborhood is sampled to extract local depth information. Median filtering is applied to eliminate depth discontinuity noise and isolate abnormal data points, ensuring reliable depth values for subsequent processing. Leveraging the intrinsic parameters from camera calibration, the framework utilizes OpenCV and PCL (Point Cloud Library) to transform the 2D skeleton feature points’ depth information into 3D coordinates within the camera coordinate system. The accuracy of this mapping is validated through a 3D point cloud visualization interface, as demonstrated in Figure 16.

Following cross-modal mapping, the 2D weld skeleton feature points are accurately reconstructed as 3D spatial trajectories within the point cloud, generating a localized, semantic-labeled point cloud region. This mapping mechanism effectively aligns 2D semantic information with 3D spatial coordinates through the integrated optimization of depth perception and geometric constraints. By ensuring millimeter-level positional precision, it establishes a reliable spatial reference for subsequent welding path planning, bridging visual semantics and robotic kinematic requirements seamlessly.

### 2.2. Research on Multi-Layer, Multi-Bead Path Planning

Following the extraction of weld centerline feature points, this study introduces a multi-layer, multi-bead path planning methodology grounded in layered slicing and feature mapping principles. Initially, a smooth spatial trajectory is generated via cubic B-spline curve fitting to the centerline, ensuring kinematic feasibility for robotic execution. The equal-arc-length layered slicing algorithm is then employed to extract cross-sectional point cloud clusters, which are processed using RANSAC linear fitting to identify robust weld feature points resilient to noise and outliers. Subsequently, these feature points undergo refinement through seventh-order polynomial interpolation, enhancing their geometric continuity and adaptability to complex weld contours. A weld bead deposition strategy based on the equal-area criterion is designed to determine layer thickness and material distribution, balancing deposition efficiency and dimensional accuracy. Finally, a JSON data structure is constructed to encode groove geometry parameters and welding torch posture configurations, which are then transformed into discrete robotic control points via a calibrated spatial transformation algorithm. This process generates executable robot code for experimental validation, bridging semantic feature extraction with practical robotic motion planning.

#### 2.2.1. Spatial Curve Fitting

In welding trajectory planning, the selection of a suitable curve-fitting model needs to take into account geometric accuracy, noise resistance, and real-time requirements. The polynomial curve uses the global parameter equation $y=\sum_{k=0}^{n}a_{k}x_{k}$ to describe the trajectory and achieves optimal fitting through the least squares method (objective function $min\sum_{i=1}^{m}(y_{i}-\sum_{k=0}^{n}a_{k}x_{i}^{k}{)}^{2}$). This method has strong noise resistance robustness by allocating noise interference to the global parameter space. At the same time, the computational complexity of the polynomial model is O(n^3^) (n is the degree of the polynomial), which is significantly lower than the O(m^3^) (m is the number of segments) of the spline interpolation and is more suitable for the needs of real-time control systems.

This paper adopts cubic polynomial fitting (formula) to generate a smooth and continuous centerline trajectory from discrete feature points. By introducing the arc length parameterization method, the spatial distribution of discrete feature points is mapped to the normalized parameter domain u ∈ [0, 1], and the objective function $min\sum_{i=1}^{n}{\left\|\left.P(u_{i})-(x_{i},y_{i},z_{i})\right\|\right.}^{2}+\lambda \int_{0}^{1}{\left.\left\|P^{\prime \prime }(u)\right.\right\|}^{2}du$ is constructed, where the regularization term λ (typical value 0.1) is used to suppress the overfitting phenomenon caused by high-order noise, and the coefficient matrix is solved:

(5) $$\left\{\begin{array}{c} X=a_{0}t^{3}+a_{1}t^{2}+a_{2}t+a_{3}\\ Y=b_{0}t^{3}+b_{1}t^{2}+b_{2}t+b_{3}\\ Z=c_{0}t^{3}+c_{1}t^{2}+c_{2}t+c_{3} \end{array}\right.\;\left(t=1,2,\ldots ,K\right)$$

where $a_{0}$, $a_{1}$, $a_{2}$, $a_{3}$, $b_{0}$, $b_{1}$, $b_{2}$, $b_{3}$, $c_{0}$, $c_{1}$, $c_{2}$, and $c_{3}$ are polynomial coefficients, t is the parameters, and K is the number of points.

**Figure 17:**
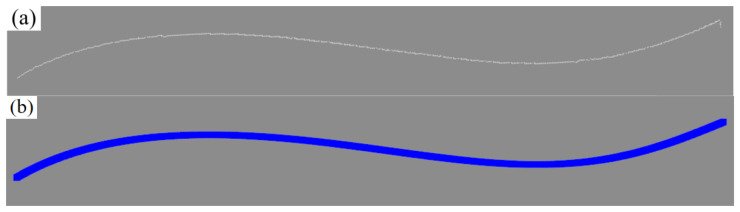
Effect of the curve-fitting algorithm. (**a**) Original point cloud; (**b**) polynomial fitting result.

When generating the section base points and their normal directions, the equal-arc-length parameterization method is used to achieve accurate geometric analysis. First, the section base points are uniformly sampled at equal arc length intervals (step length Δs = 2 mm) along the fitting trajectory, and their three-dimensional coordinates are directly calculated through parametric equations. Then, the tangent direction is determined by the first-order derivative of the trajectory, and the normal direction is made consistent with the tangent direction to meet the axial control requirements of the welding robot’s welding gun posture. The effect of the polynomial curve-fitting algorithm is shown in Figure 17.

#### 2.2.2. Welding Groove Feature Point Extraction

Accurate extraction of welding groove feature points is crucial for trajectory planning and process stability. For typical V-shaped grooves (opening angle 45° ± 5°; depth 8–12 mm), this paper proposes an improved RANSAC method for 3D point cloud section feature recognition. The method enhances noise resistance and fitting accuracy by introducing dynamic parameter adjustment, multi-stage geometric constraints, and centroid clustering sampling.

**Figure 18:**
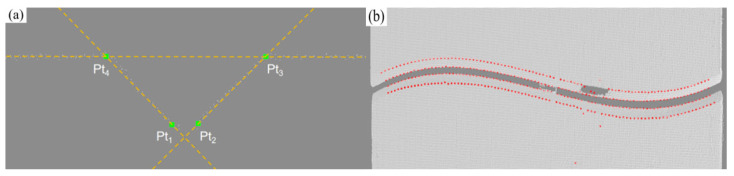
Welding groove feature point extraction. (**a**) Line fitting; (**b**) feature point extraction results.

The algorithm dynamically adjusts the inlier threshold to handle local deformation and adapts the maximum number of iterations based on point cloud scale for efficient, robust line fitting. The parent material plane, two side bevels, and bottom feature points are extracted, with key points (Pt_1_~Pt_4_) located by intersection and extreme points. Geometric rule verification is applied to eliminate abnormal points and verify groove width and opening angle rationality, improving feature point stability and process adaptability. The typical position extraction effect is shown in Figure 18.

**Figure 19:**
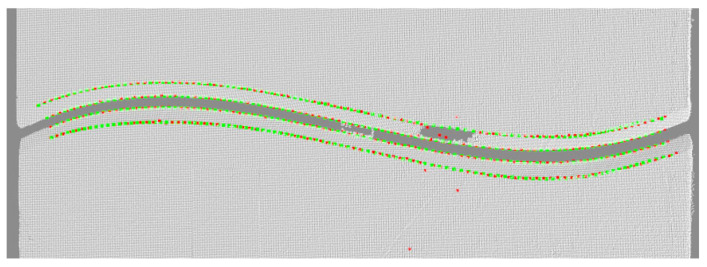
Seventh-order polynomial fitting optimization results.

Finally, a seventh-order polynomial is used to perform global fitting optimization on the feature point sequence to achieve feature point reconstruction with millimeter-level accuracy. As shown in Figure 19 this method shows good stability and accuracy in the actual point cloud, with a vertex positioning error (MAE) of 0.238 mm and a bottom point of 0.269 mm, which verifies the reliability of the method.

#### 2.2.3. The Scheme of Multi-Layer and Multi-Channel Path Planning

To achieve high-quality welding of V-shaped grooves, path planning involves three key tasks: first, formulating a weld bead filling strategy to ensure consistent cross-section shape and improve stability; second, accurately planning the welding gun’s spatial posture to match the groove’s geometric contour; and third, using affine transformation and stereo vision data to map the path from the 2D model to the 3D point cloud, adapting to groove size changes and supporting dynamic path generation.

**Figure 20:**
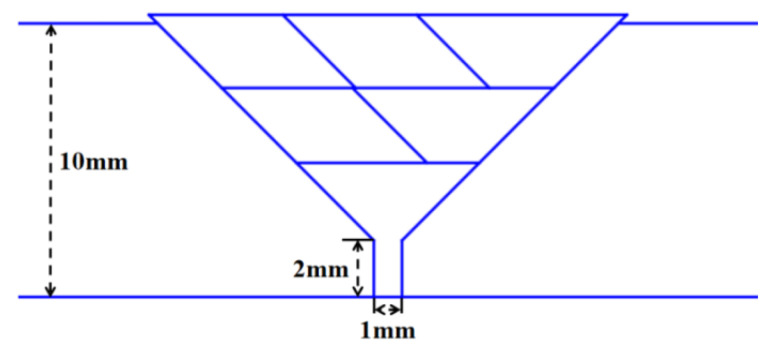
Weld bead filling scheme based on equal area method.

This paper proposes a weld bead filling strategy based on the equal area method, following the principles of “layered equal number of passes” and “consistent cross-sectional area” to ensure stable weld formation. The filling order consists of a trapezoidal cross-section for the first layer, a parallelogram for the middle layer, and a trapezoid for the last layer, creating a ladder structure. The planning strategy is shown in Figure 20.

For process parameters, standard conditions (current 200 A; voltage 22 V; travel speed 6.0 mm/s) are used as references, with a standard weld bead area of 13.0 mm^2^ to minimize parameter adjustments and improve consistency. During welding, the “fixed as the main, fine-tuning as auxiliary” strategy keeps main parameters stable, while the stereo vision system dynamically monitors the groove, adjusts the travel speed, and cooperates with the high-dynamic camera to observe the molten pool, ensuring welding quality.

The welding gun posture is defined by the position coordinates (x, y, and z) and the posture angle (α, β, and γ), which represent the spatial position of the welding gun tip and the rotation angle around the X-, Y-, and Z-axes, respectively.

**Figure 21:**
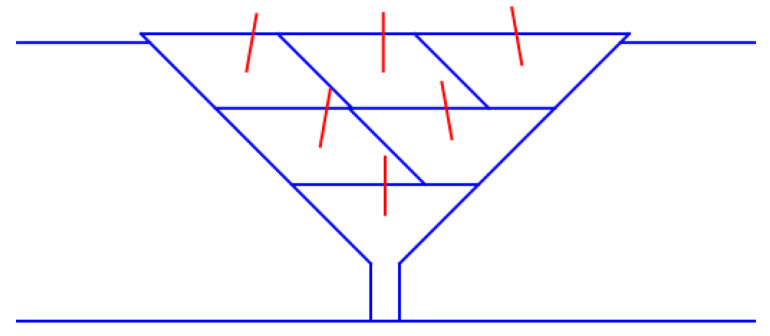
Weld bead orientation planning results.

This study integrates geometric modeling and kinematic algorithms to compute the arc start position and torch posture for welds. For parallelogram-shaped weld geometries, the welding torch traverses along the perpendicular bisector of the long diagonal, a strategy that ensures weld formation by balancing heat input distribution across the joint. In the case of trapezoidal welds, center line positioning is employed to maintain alignment with the cross-sectional centroid, a critical geometric property that enhances dimensional uniformity by minimizing off-center material deposition and ensuring consistent bead profiles. These orientation planning strategies for weld beads are visually illustrated in Figure 21, which presents the weld bead orientation planning results and complements the theoretical description with graphical representation.

The determination of torch posture angles must account for multiple critical factors, including collision avoidance, bead formation quality, and penetration depth. For instance, in experiments involving 45° or 60° V-groove welds, rotating the welding torch by 10° around the X-axis (α = 10°) mitigates mechanical interference while ensuring adequate fusion and stable welding quality. This angular adjustment balances geometric constraints of the groove with kinematic requirements, demonstrating a systematic approach to posture optimization that prioritizes both process reliability and structural integrity.

Subsequent to defining the welding torch posture, the planar standard model is mapped to the 3D groove point cloud to enable precise path adaptation across geometric transitions. The fundamental principle of the spatial mapping algorithm involves establishing a geometric correspondence between the planar reference model and groove feature points, facilitating the generation of a welding trajectory that aligns with the groove’s actual geometric morphology.

In welding, the groove feature points (Pt_1_~Pt_4_) are located in the 3D space, and the plane model consists of 2D feature points A, B, C, and D. To establish the correspondence, this paper uses affine transformation to map the 2D feature points to the 3D target points through rotation, scaling, shearing, and translation operations.

The affine transformation matrix and displacement vector are solved by establishing a one-to-one correspondence between the 2D points (A, B, C, and D) and 3D points (Pt_1_~Pt_4_). The affine transformation equation is substituted into a linear system, which is then solved using the least squares method to obtain the optimal affine transformation matrix and displacement vector.

**Figure 22:**
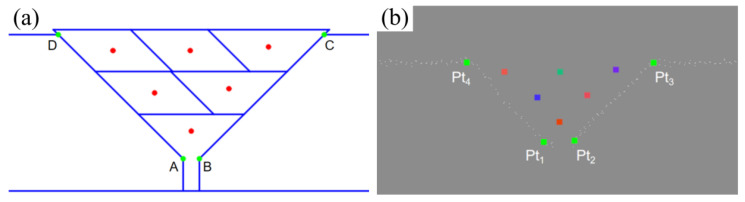
Spatial mapping algorithm effect. (**a**) Plane standard model; (**b**) 3D point cloud spatial mapping result.

After solving for the mapping parameters, the welding gun posture in the plane model can be mapped to the groove’s 3D coordinate system. Using the affine matrix and displacement vector, the 2D point is converted to a 3D point, and the corresponding attitude angle is calculated to obtain the 3D position and orientation for the actual welding path. As shown in Figure 22, the planned path on the plane and the corresponding path points mapped into 3D space are illustrated, respectively.

In addition, to improve path adaptability, the system introduces a cross-sectional area adaptation mechanism: the system uses stereo vision to monitor the difference between the current groove cross-sectional area S_actual_ and the standard value S_standard_ = 13.0 mm^2^. If the threshold is exceeded, the speed compensation mechanism is triggered to automatically adjust the travel speed to avoid excessive metal accumulation or unfused problems.

(6) $$v_{actual}=v_{standard}\times S_{standard}/S_{actual}$$

In summary, the algorithm achieves accurate conversion from the plane path to the 3D point cloud space, taking into account the adaptability of groove geometry changes and path planning efficiency. Taking three-layer six-pass welding as an example, about 600 sets of welding gun postures can be automatically generated, greatly improving the welding path accuracy and system response speed and significantly reducing manual intervention and planning time.

## 3. Experimental Verification

**Figure 23:**
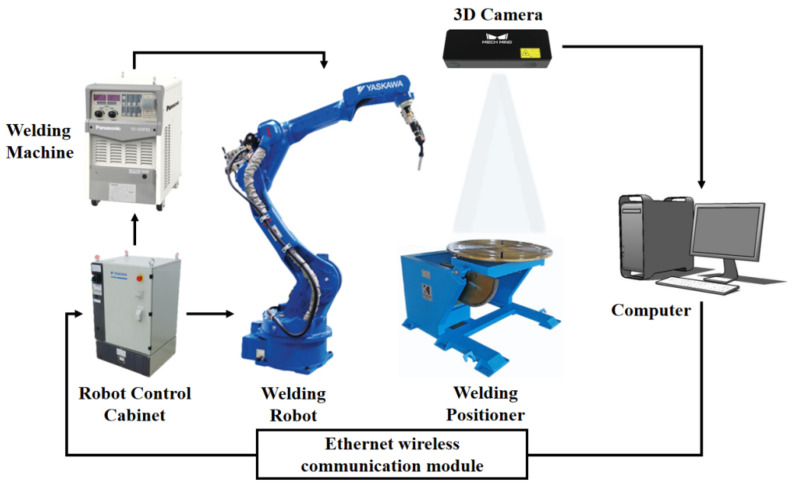
System composition schematic diagram.

The hardware system comprises a Motoman MH24 six-axis industrial robot that is manufactured by Yaskawa in Kitakyushu, Japan, a rotation-tilt positioner, a Panasonic YD-500FR welding power source produced by Panasonic in Osaka, Japan, a Mech-Eye Pro M Enhanced industrial-grade 3D camera developed by Mech-Mind in Beijing, China, and a host computer, as illustrated in Figure 23. Functionally, the system is divided into two integrated subsystems: the robotic welding system and the stereo vision sensing system. The stereo vision sensing system acquires 2D images and 3D point cloud models of the workbench surface topography, transmitting these data to the host computer for processing. Conversely, the robotic welding system executes programmed motions, guiding the welding torch along preplanned trajectories while adjusting the process parameters to ensure precise weld deposition.

The eye-to-hand system configuration was selected, and the Mech-Eye Pro M Enhanced industrial-grade 3D camera was installed externally on the top of the working space and fixed independently of the robot body with a rigid bracket. The device integrates DLP structured light active projection technology and realizes high-precision 3D imaging through coded grating fringe projection. It can simultaneously collect 2D images (1920 × 1200 resolution), depth information, and high-density 3D point cloud data. Its effective working distance is 1000–2000 mm, and it achieves the VDI/VDE certified 0.2 mm spatial accuracy at a measuring distance of 2 m. During the experiment, the camera quickly captured the surface morphology of the working area and realized the full-scene 3D modeling of the workbench through the point cloud data processing algorithm, providing a sub-millimeter spatial benchmark for subsequent weld geometry feature extraction and path planning.

**Figure 24:**
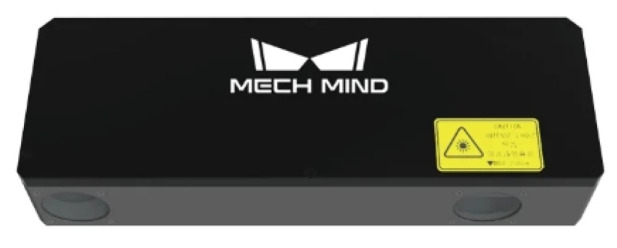
Mech-Eye camera, actual picture.

**Figure 25:**
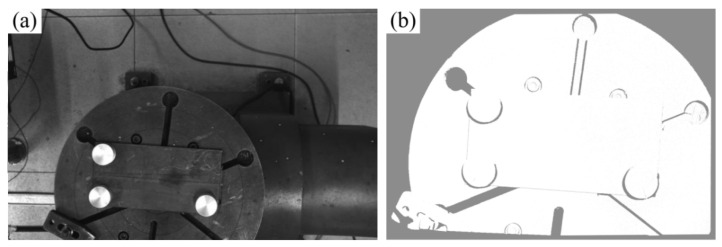
Mech-Eye camera data acquisition format: (**a**) 2D image; (**b**) 3D point cloud.

This paper uses randomly placed flat-plate V-butt welds as the verification object and verifies the weld intelligent recognition and path planning algorithm developed in this paper through full-process automated testing. The experiment adopts a hierarchical progressive verification strategy: first verifying the system accuracy, then evaluating the accuracy of the welding gun path planning, and finally verifying the system performance through actual welding. The actual camera image and the collected image and point cloud are shown in Figure 24 and Figure 25, respectively.

### 3.1. Hand–Eye Relationship Calibration and System Accuracy Verification Experiment

**Figure 26:**
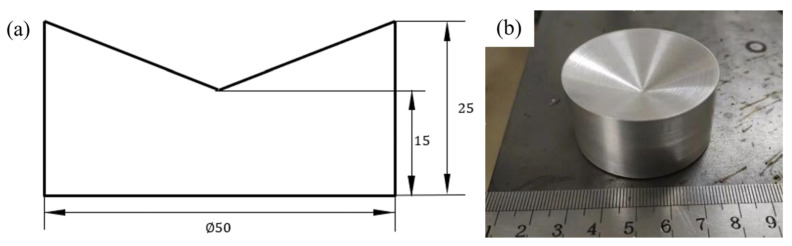
Dimensions and photograph of the calibration block. (**a**) Calibration block dimensions; (**b**) calibration block photograph.

**Figure 27:**
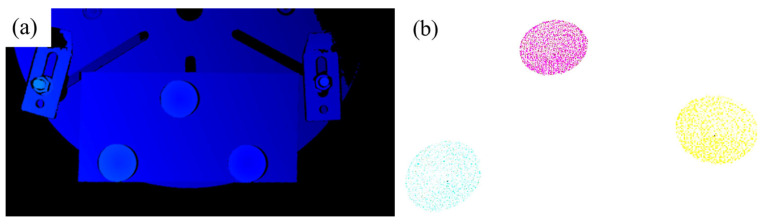
Extraction of calibration block point clouds. (**a**) Captured point cloud; (**b**) point cloud filtering and clustering.

This paper directly calibrates the camera coordinate system and user coordinate system of the point cloud. By simultaneously constructing the corresponding user coordinate system (UCS) in the model and the robot system, the position matching between the program path and the point cloud is achieved. To more accurately find feature points in the point cloud model, a calibration block processed from a cylinder with a concave conical surface on the upper surface was specially designed in order to extract the center point through cone fitting on the point cloud, and the structure is shown in Figure 26. The specific operation process is as follows:

(1) First, place 3 calibration blocks on the additive manufacturing test plate, and use a 3D camera to capture the point cloud model of the upper surface morphology of the calibration blocks, that is, a conical surface. Subsequently, fit this conical surface to obtain the conical vertex coordinates (the obtained point cloud model is shown in Figure 27a). Next, use pass-through filtering to extract the three calibration blocks, then perform processing such as voxel downsampling and statistical filtering on them, and separate the calibration blocks through Euclidean clustering. The final conical point cloud of the calibration blocks is shown in Figure 27b).

(2) Let P be the conical vertex, V be any point on the conical surface, $\vec{u}$ be the unit vector of the conical main axis direction vector, and θ be the semi-apex angle of the cone. Then, the equation of the conical surface can be expressed as Formula (7).

(7) $$\left(P-V\right)\cdot \vec{u}=\left\|P-V\right\|\cdot cos\theta$$

**Figure 28:**
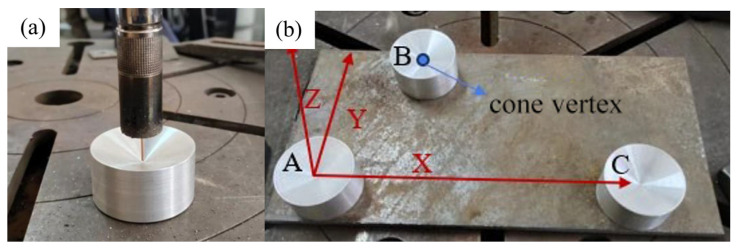
Calibration of user coordinate system in robot system. (**a**) Teaching process; (**b**) schematic of user coordinate system.

The Random Sample Consensus (RANSAC) algorithm is used to iteratively fit the cylindrical surface, so as to effectively and accurately extract the conical vertex coordinates, even in the presence of noise points. The conical vertices of the three fitted calibration blocks are, respectively, marked as *P*_A_, *P*_B_, and *P*_C_. Taking the first point *P*_A_ as the origin, the vector is determined as the X-axis direction of the user coordinate system. The Z-axis direction is determined by the cross-product of $\left|\vec{P_{A}P_{C}}\right|$ and $\left|\vec{P_{A}P_{B}}\right|$, and then the Y-axis direction is obtained through the cross-product of the Z- and X-axis vectors, thus successfully establishing the user coordinate system in the point cloud. Similarly, in the robot system, with the help of the teaching method, the three-point method is used to establish the corresponding user coordinate system, and the specific situation is shown in Figure 28.

To verify the calibration accuracy of the system, after the calibration is completed, the conical calibration blocks are placed at different positions as checkpoints. The methods of robot tool point touch and camera shooting point cloud and vertex fitting, respectively, are used to obtain the coordinates of the same point in the user coordinate system established in the model and the user coordinate system in the robot system, and the difference between the two is calculated. The specific data are shown in Table 2. From the final results, it can be seen that the average calibration error is within 0.5 mm, basically meeting the accuracy requirements for GMA additive manufacturing.

**Figure 29:**
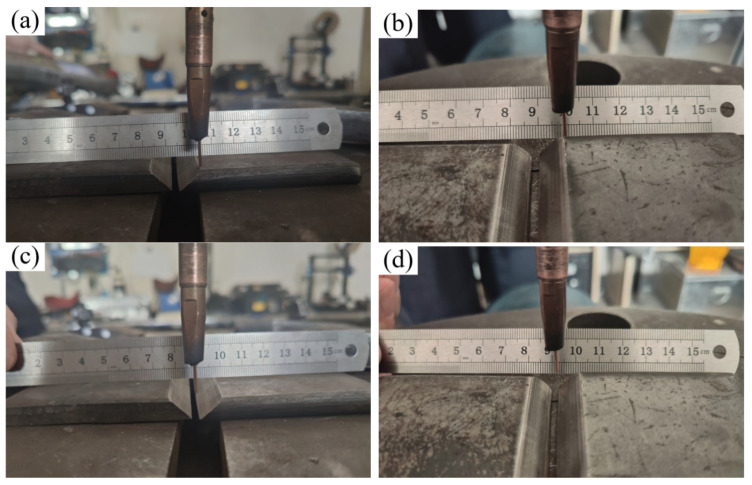
Welding gun posture during robot operation. (**a**) Initial position of weld edge; (**b**) end position of weld edge; (**c**) initial position of weld; (**d**) end position of weld.

The ultimate goal of this system is to perform feature extraction based on information acquired by a 3D camera (including images and point clouds) and to plan the robotic welding path accordingly. The system’s overall error mainly originates from three aspects: (1) errors in data acquisition by the camera, including point cloud inaccuracies and the mapping error between image pixels and point cloud points; (2) calibration errors in the hand–eye relationship between the camera and the robot; and (3) intrinsic errors in the robotic system, such as motion accuracy and the precision of the tool coordinate system. This study evaluates system accuracy through experimental validation on a straight V-groove workpiece. First, the camera captures the scene, and the proposed algorithm is used to extract the V-groove edge features. These features are then transformed into the robot coordinate system using the calibrated hand–eye relationship. The robot is subsequently guided to move to the corresponding positions, and the deviation between the actual and target positions is measured using a caliper, thereby assessing the system’s precision. The proposed algorithm enables the system to autonomously guide the robot to the extracted weld centerline and plate edge positions, and the results are shown in Figure 29.

**Figure 30:**
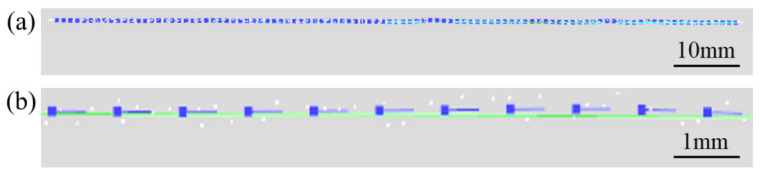
Comparison between the weld plate edge trajectory generated by the system and the manual teaching line. (**a**) Overall; (**b**) details.

**Figure 31:**
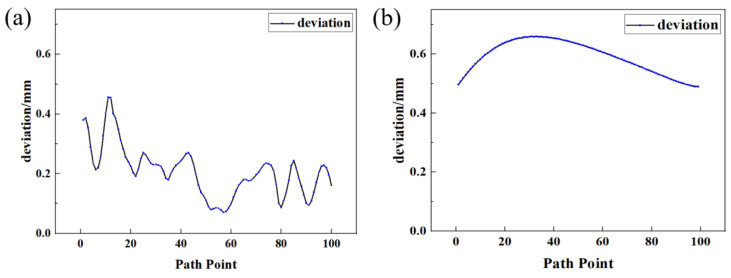
Deviation distribution between the system-generated trajectory and the manually taught line. (**a**) Welding plate edge trajectory; (**b**) welding gun movement trajectory.

In order to verify the accuracy of the system, the generated weld plate edge trajectory was compared with the edge position obtained through teaching, and the deviation was analyzed. The blue discrete points in Figure 30 are the path points of the weld plate edge trajectory generated by the system, and the green line is the weld plate edge position obtained through manual teaching. The spatial position deviation distribution between the two is shown in Figure 31. The calculated average deviation is 0.208 mm, and the mean square error is 0.224 mm.

In order to evaluate the planning accuracy of the welding gun motion trajectory, the flat V-shaped weld path generated through manual teaching is used as the standard model, and the generated welding gun motion trajectory is compared and analyzed for deviation. The spatial position deviation distribution between the two is shown in Figure 26b. The calculated average deviation is 0.590 mm, and the mean square error is 0.593 mm.

The results show that the trajectory generated by the stereo vision weld autonomous recognition system is equivalent to the manual teaching effect and meets the requirements of general welding.

### 3.2. Multi-Layer, Multi-Bead Verification Experiment

This experiment uses curved welds as the object to verify the full-process automation performance of the system under complex backgrounds, including weld autonomous identification, multi-layer, multi-bead path planning, and dynamic welding control capabilities.

The weld contour is segmented by improving the U-Net model, and 100 feature points are generated through skeleton refinement and dynamic equidistant sampling; then, the weld centerline is fitted with a cubic polynomial curve, 100 cutting planes are generated equidistantly along the path, and groove feature points (Pt_1_~Pt_4_) are extracted; finally, the preset three-layer six-pass weld filling scheme is mapped to three-dimensional space using a spatial mapping algorithm to generate welding gun trajectory and posture data, as shown in the figure.

The system generates *.JBI files to drive the Motoman robot to perform welding tasks. The system adjusts the travel speed in real time to compensate for the fluctuation of the groove cross-sectional area and integrates a high-dynamic camera to capture the molten pool shape in real time.

**Figure 32:**
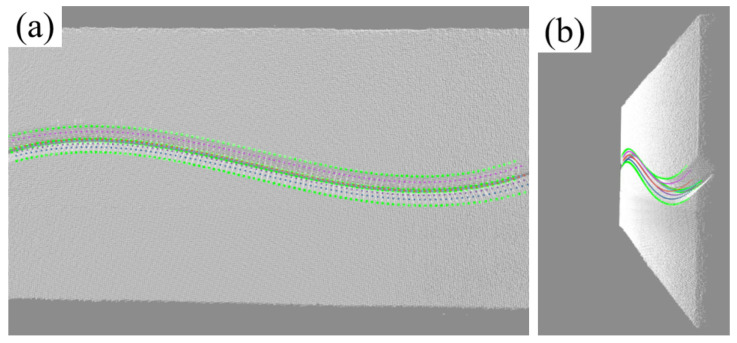
Multi-layer, multi-bead welding gun trajectory planning results. (**a**) Front view (with reference track line); (**b**) side view (with reference track line).

**Figure 33:**
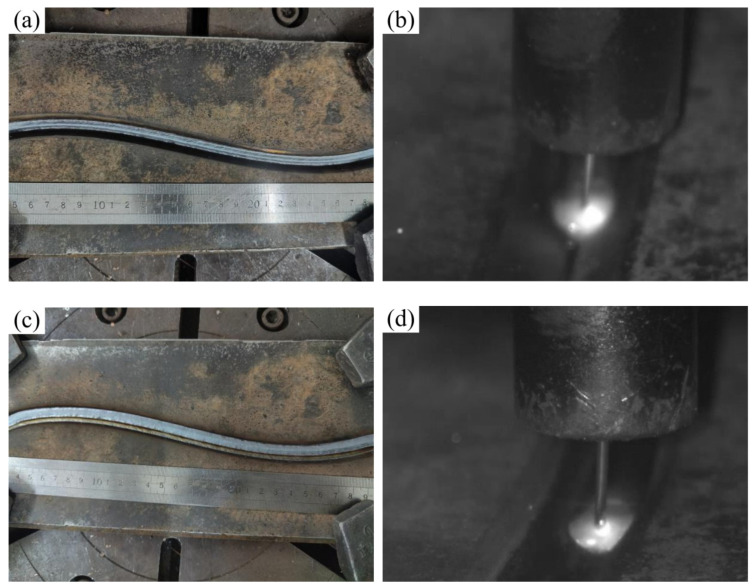

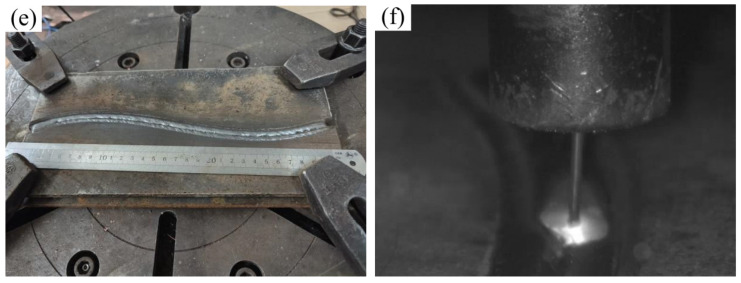
Welding results and molten pool morphology. (**a**) Welding result of the first layer (one pass); (**b**) shape of the first molten pool; (**c**) welding result of the second layer (three passes); (**d**) shape of the third molten pool; (**e**) welding result of the third layer (six passes); (**f**) shape of the sixth molten pool.

The effect of feature point recognition and path planning on the point cloud is shown in Figure 32, and the welding process and results are shown in Figure 33. Experiments show that the system can achieve coordinated optimization of cladding uniformity (melt width fluctuation ≤ ±0.15 mm), process stability, and forming quality in curved multi-layer, multi-bead welding scenarios, meeting the process requirements of multi-layer welding. The high dynamic molten pool camera can monitor the welding process. The molten pool image and post-welding results show that the welding quality is well.

**Figure 34:**
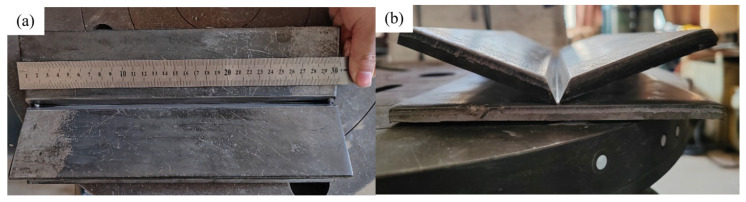
Weld seam photos: (**a**) front view; (**b**) side view.

**Figure 35:**
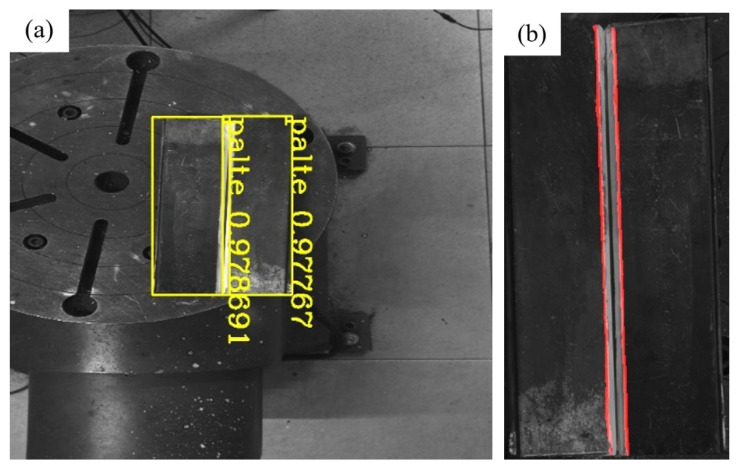
Deep learning network recognition results. (**a**) YOLOv8 model recognition of weld plate results; (**b**) improved U-Net model recognition of weld boundary results.

**Figure 36:**
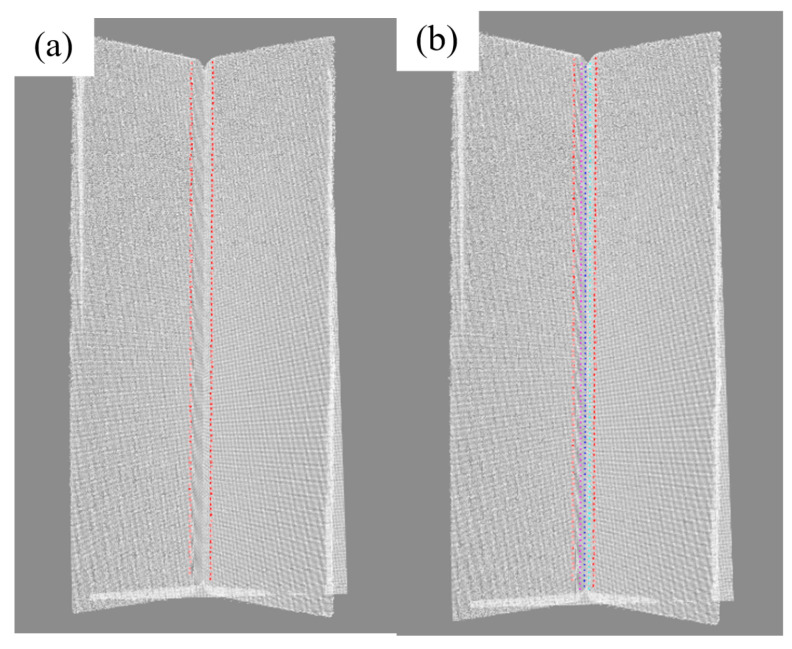
Weld groove feature recognition and multi-layer and multi-bead weld arrangement. (**a**) Boundary feature point recognition; (**b**) multi-layer and multi-bead weld arrangement.

**Figure 37:**
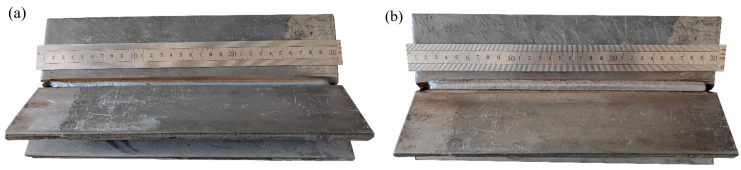
Fillet weld results: (**a**) after the first weld bead; (**b**) after the third weld bead.

To further verify the applicability of the system, a V-groove fillet weld was selected for validation. The weld geometry is shown in Figure 34. Figure 35 presents the results of weld plate detection using the YOLOv8 model and weld boundary extraction using the improved U-Net model, demonstrating the accurate identification of both the weld plate and the weld boundary. Figure 36 illustrates the groove feature points obtained through curve fitting and the multi-layer multi-pass path planning results, with a total of three welding passes planned. The welding process and final outcome are shown in Figure 37. The results of both experiments verify that the proposed method can achieve high-precision weld identification and stable path planning under certain working conditions, demonstrating strong robustness and feasibility.

## 4. Conclusions

Aiming at the task of the generation of robot welding trajectories, this paper proposes a dual-modal collaborative method of “2D image autonomous recognition + 3D point cloud precise planning” that integrates stereo vision and deep learning and realizes high-precision recognition and path planning of welds under complex working conditions. In terms of 2D image recognition, a dataset containing straight and curved welds is constructed, a fast-welding, plate-positioning method based on YOLOv8 is proposed, and an improved U-Net is designed. The dual-channel attention mechanism and hybrid loss function are introduced to greatly improve the weld segmentation performance, with the mIoU increasing by 11.4% (to 0.887), precision increasing from 0.875 to 0.937, and recall increasing from 0.895 to 0.944. The skeleton extraction and uniform interpolation method are further combined to achieve sub-millimeter feature point precise extraction. In terms of 3D point cloud modeling and trajectory planning, 2D semantic information is mapped to the 3D point cloud, key feature points of groove are extracted via layered slicing and improved RANSAC, and smooth trajectory is generated via seventh-order polynomial fitting; at the same time, a weld bead filling method based on the equal area strategy is proposed, the dynamic mapping of standard weld bead model in different grooves is realized by combining affine transformation, and the multi-axis posture parameters of welding gun are accurately derived, so as to complete multi-layer, multi-bead path planning. The experimental results verify the excellent performance of the system in terms of stability and accuracy: the average trajectory deviation of straight weld is 0.229 mm, the three-axis trajectory deviation of welding gun is 0.590 mm, and the overall trajectory accuracy is controlled within 0.7 mm; in complex curve welding scenes, the fluctuation of weld width is less than ± 0.15 mm, which proves that the method in this paper can achieve high-precision weld recognition and stable path planning under complex working conditions and has good robustness and feasibility.

In the future, multi-sensor information, deep reinforcement learning, and neural network technology can be further integrated to improve the system’s adaptability and intelligence level to complex 3D welds. For example, the combination of structured light vision and a target tracking algorithm can improve the positioning accuracy of weld feature points and enhance the robustness of target detection in external interference environments, thereby further improving the accuracy and stability of path planning.

## Figures and Tables

**Table 1:** Network performance on the test set.

Models (Backbone)	mIoU	MPA	Cost Time (ms)
Improved U-Net (VGG16)	87.54	95.4	105.8
pspNet (Resnet50)	70.28	86.3	147.3
DeepLab V3+ (Xception)	75.67	90.4	203.7

**Table 2:** Accuracy verification.

No.	UCS in Camera (mm)	UCS in Robot System (mm)	Error (mm)
1	(61.124, 82.098, 0.056)	(61.074, 82.347, −0.239)	(0.050, −0.249, 0.295)
2	(210.366, 91.349, −0.017)	(209.975, 91.598, −0.054)	(0.391, −0.249, 0.037)
3	(68.375, 154.357, 0.139)	(68.633, 154.511, 0.210)	(−0.258, −0.154, −0.071)
4	(220.311, 169.564, 5.566)	(220.008, 169.858, 5.430)	(0.303, −0.294, 0.136)

## Data Availability

The original contributions presented in this study are included in the article. Further inquiries can be directed to the corresponding author.

## References

[1] Wang T., Li Q., He J. (2024). A precise guiding technology for V-groove initial weld point based on binocular vision and structured light vision. Int. J. Adv. Manuf. Technol..

[2] Mu S., Wang J., Mu C. (2024). The Chaos Sparrow Search Algorithm: Multi-layer and multi-pass Welding Robot Trajectory Optimization for Medium and Thick Plates. J. Bionic Eng..

[3] Li G., Hong Y., Gao J., Hong B., Li X. (2020). Welding seam trajectory recognition for automated skip welding guidance of a spatially intermittent welding seam based on laser vision sensor. Sensors.

[4] Xu Y., Wang Z. (2021). Visual sensing technologies in robotic welding: Recent research developments and future interests. Sens. Actuators A Phys..

[5] Xu F., Xu Y., Zhang H., Chen S. (2022). Application of sensing technology in intelligent robotic arc welding: A review. J. Manuf. Process..

[6] Wang B., Hu S.J., Sun L., Freiheit T. (2020). Intelligent Welding System Technologies: State-of-the-Art Review and Perspectives. J. Manuf. Syst..

[7] Wang Z., Xu Y. (2020). Vision-Based Seam Tracking in Robotic Welding: A Review of Recent Research.

[8] Zhou P., Peng R., Xu M., Wu V., Navarro-Alarcon D. (2021). Path Planning with Automatic Seam Extraction Over Point Cloud Models for Robotic Arc Welding. IEEE Robot. Autom. Lett..

[9] Zhang G., Zhang Y., Tuo S., Hou Z., Yang W., Xu Z., Wu Y., Yuan H., Shin K. (2021). A Novel Seam Tracking Technique with a Four-Step Method and Experimental Investigation of Robotic Welding Oriented to Complex Welding Seam. Sensors.

[10] Chi D., Wang Z., Liu H. (2024). Automatic Defects Recognition of Lap Joint of Unequal Thickness Based on X-Ray Image Processing. Materials.

[11] Lei T., Gong S., Wu C. (2024). A Multi-Layer Multi-Pass Weld Bead Cross-Section Morphology Extraction Method Based on Row–Column Grayscale Segmentation. Materials.

[12] Zhang X., Zhao S., Wang M. (2024). Deep Learning-Based Defects Detection in Keyhole TIG Welding with Enhanced Vision. Materials.

[13] Liu H., Wu Y., Chen Z. (2024). Penetration State Recognition during Laser Welding Process Control Based on Two-Stage Temporal Convolutional Networks. Materials.

[14] Malta A., Mendes M., Farinha T. (2021). Augmented Reality Maintenance Assistant Using YOLOv5. Appl. Sci..

[15] Bist R.B., Subedi S., Yang X., Chai L. (2023). A Novel YOLOv6 Object Detector for Monitoring Piling Behavior of Cage-Free Laying Hens. AgriEngineering.

[16] Wang C.Y., Bochkovskiy A., Liao H.Y.M. YOLOv7: Trainable Bag-of-Freebies Sets New State-of-the-Art for Real-Time Object Detectors. Proceedings of the 2023 IEEE/CVF Conference on Computer Vision and Pattern Recognition (CVPR).

[17] Varghese R., Sambath M. YOLOv8: A Novel Object Detection Algorithm with Enhanced Performance and Robustness. Proceedings of the 2024 International Conference on Advances in Data Engineering and Intelligent Computing Systems (ADICS).

[18] Yang Y., Yang Y., Shao W. (2025). Micro-Gap Weld Seam Contrast Enhancement via Phase Contrast Imaging. Materials.

[19] Ronneberger O., Fischer P., Brox T. (2015). U-Net: Convolutional Networks for Biomedical Image Segmentation. International Conference on Medical Image Computing and Computer-Assisted Intervention.

[20] Fu J., Liu J., Tian H., Li Y., Bao Y., Fang Z., Lu H. Dual Attention Network for Scene Segmentation. Proceedings of the 2019 IEEE/CVF Conference on Computer Vision and Pattern Recognition (CVPR).

[21] Li X., Sun X., Meng Y., Liang J., Wu F., Li J. Dice Loss for Data-imbalanced NLP Tasks. Proceedings of the 58th Annual Meeting of the Association for Computational Linguistics.

[22] Lin T.Y., Goyal P., Girshick R., He K., Dollár P. Focal Loss for Dense Object Detection. Proceedings of the 2017 IEEE International Conference on Computer Vision (ICCV).

[23] Cho Y.J. (2024). Weighted Intersection over Union (wIoU) for evaluating image segmentation. Pattern Recognit. Lett..

